# An exploration of status of chronic diseases and its influencing factors of older people in Chinese home care and long-term care facilities: a cross-sectional study

**DOI:** 10.3389/fpubh.2023.1321681

**Published:** 2023-12-21

**Authors:** Fen Xie, Qingxia Shu, Jinxiu Li, Zheng-ying Chen

**Affiliations:** ^1^School of Nursing and Midwifery, Trinity College Dublin, Dublin, Ireland; ^2^Jishou University School of Medicine, Jishou, China; ^3^Hunan University of Medicine General Hospital, Huaihua, China

**Keywords:** older people, home care, long term care facilities, chronic disease, influencing factors

## Abstract

**Background:**

As the population ages, the proportion of chronic diseases becomes more prevalent. This study aimed to investigate the current status of chronic diseases among the older people in home care (HC) and long-term care facilities (LTCFs) in China and to analyze its influencing factors.

**Methods:**

This cross-sectional study was conducted between 2021 and 2022. A multi-stage stratified random sampling and census sampling approach was used in this survey of the health of 389 older people in HC and 202 older people in LTCFs from Western Hunan, respectively. The following instruments were included in the survey “International Resident Assessment Instrument for Home Care (interRAI-HC)” and the “International Resident Assessment Instrument for Long-Term Care Facilities (interRAI-LTCF).” Univariate analysis was used to examine the prevalence of chronic diseases among older people with different characteristics. Data were analyzed by IBM SPSS version 25.0 software. A *p*-value of <0.05 was considered statistically significant.

**Results:**

The survey results showed that the prevalence of chronic diseases among older people in HC was 73.26% (95% CI, 68.85–77.68), and the top five chronic diseases were hypertension (26.36%), bone and joint disease (23.36%), gastrointestinal and gallbladder disease (11.78%), heart disease (11.21%), and diabetes (8.97%). The prevalence of chronic diseases among older people in LTCFs was 77.23% (95% CI, 77.23–83.06), and the top five chronic diseases were hypertension (33.11%), bone and joint disease (13.25%), cerebrovascular disease (12.91%), diabetes (11.26%), and heart disease (10.26%). The results showed that long time spent alone, having sleep disorders, and self-rated health status significantly increased HC in older people with the prevalence of chronic diseases (*p* < 0.05). Having marital status, non-healthy BMI, having sleep disorder, walking with the use of assistive devices, and self-rated health status significantly increased older people in LTCFs with the prevalence of chronic diseases (*p* < 0.05).

**Conclusion:**

There are differences in the prevalence and distribution of chronic diseases among older people in two different aged care models in China, and there are various risk factors for chronic diseases. Therefore, chronic disease healthcare strategies should be tailored to two different aged care models for older people. Further summary found that older people in HC spend a lot of time alone and suffer from loneliness, which ultimately causes psychological disorders. Thus, psychological adaptation interventions are needed for older people in HC. Besides, older people in LTCFs lack social support from their families (divorced/widowed) and have activity disorders (walking with the use of assistive devices). Thus, social adaptation interventions are needed for older people in LTCFs. This study provides a theoretical basis for the distribution of healthcare and the prevention and treatment of chronic diseases in Chinese older people.

## 1 Introduction

Population aging is one of the major trends in socio-demographic development, and it has become a major public health issue in China. China has the largest older population in the world (1). The older population aged 60 years and above account for 18.70% (264 million) of the total population in 2021 (2) and will enter the stage of severe aging in 2035 (3). The sudden increase in the older population causes the fact that older people are not only the main group of people suffering from chronic diseases, but they are also the main people group dying as a result of chronic diseases. The World Health Organization reported that chronic diseases account for 73.60% of all deaths worldwide (4), and such deaths are mainly concentrated in developing countries (5). Therefore, there is an urgent need to reduce and delay the prevalence of chronic diseases among older people in China.

Nursing healthcare is an effective method for reducing and delaying the prevalence of chronic diseases in the older people. However, it is based on the theoretical premise of analyzing and obtaining the key influencing factors of chronic diseases. Thus, key influencing factors of chronic diseases are urgently needed to be studied. Studies showed that chronic diseases can be prevented and intervened as early as possible, thus increasing life expectancy and reducing the incidence of major chronic diseases (6, 7). Ranjbaran surveyed 734 patients with type 2 diabetes and found that determinants of diet (8) and medication (9) adherence could be identified based on the health action process approach. The intervention was further designed based on obtained determinants and was found to effectively improve the diet and medication adherence of patients (10). In addition, perceived social support can improve patients’ quality of life and overcome cardiovascular diseases (11). Moreover, intervention measures based on PRECED-PROCED model can effectively improve the sleep quality of patients after coronary artery bypass grafting (12). May reported that older people in Netherlands with healthy lifestyles live longer (13). Danaei and Stanaway reported that being physically active, maintaining a normal body weight, avoiding smoking, and drinking moderately reduce the risk of total mortality and chronic diseases (14, 15). In contrast, lack of exercise (16), unhealthy diet (17), smoking (18), excessive alcohol consumption (19), and obesity (20) increase the risk of chronic diseases.

To respond to the problem of the aging population and chronic diseases, the Chinese government is currently aiming to develop long-term care (LTC) on the basis of home care (HC). However, there is currently a lack of sufficient understanding of chronic diseases among older people in two different aged care models in China. Thus, this study aimed to explore the status of chronic diseases in older people in Chinese home care and long-term care facilities (LTCFs). Through a systematic investigation of the current status of chronic diseases among the older people under different aged care models, chronic disease influencing factor models were developed. This study provides theoretical guidance for the development of appropriate healthcare strategies for older people.

## 2 Methods

### 2.1 Study design and participants

This cross-sectional study was conducted between 2021 and 2022. The older people in HC was based on the number of older population (*N* = 13.21 million) drawn from the seventh national population census of Hunan Province (21), of which 192,096 were in the urban areas of Western Hunan. The confidence level and sampling error were set as 95 and 5%, respectively. Thus, the sample size is 385 in this survey study. Using a multi-stage stratified random sampling method, three cities were selected from Western Hunan, three streets were randomly selected from each of the sample cities (a total of nine streets), and one community was randomly selected from each sample street (total of nine communities). At last, 30–50 cases of older people in HC randomly selected from each sample community were surveyed.

A census sampling approach was used to investigate the health of older people aged 60 years (*n* = 202) in all long-term care facilities (*n* = 12) from urban areas of Western Hunan. (1) Inclusion criteria: age ≥ 60 years and agreement to participate. (2) Exclusion criteria: people who are unconscious or unable to communicate and leave HC and LTCFs during the research period. The study was approved by the Institutional Ethics Committee and the Biomedical Ethics Committee. Additionally, all respondents were informed of the study objectives and an informed consent was obtained. Therefore, the final sample consisted of 389 older people in HC and 202 older people in LTCFs.

### 2.2 Research methods

The following instruments were included in the survey “International Resident Assessment Instrument for Home Care (interRAI-HC)” and the “International Resident Assessment Instrument for Long-Term Care Facilities (interRAI-LTCF).” The interRAI-HC and interRAI-LTCF Assessment Scale includes basic information and several dimensions of assessment: cognition, communication and vision, psychosocial wellbeing, functional status, disease diagnosis, and health conditions. These scales have been widely used around the world in more than 40 countries and regions. Moreover, they have been used to evaluate the care needs and health problems of older people, with high reliability and validity (22–25).

After obtaining the consent of the respondents, surveyors conducted face-to-face interviews using paper questionnaires, assisted by institutional management, nursing staff, or family members. The questionnaires were collected on-site and reviewed by two surveyors in order to add missing items as soon as possible.

### 2.3 Statistical analysis

The data entry and statistical analysis were performed using IBM SPSS Statistics for Windows version 25.0 (IBM Corp., Armonk, NY, United States). The measured data are expressed as mean ± standard deviation. Count data are expressed as proportion ratios or rates and 95% confidence intervals. Univariate analysis will be used to examine whether there are differences in the prevalence of chronic diseases among older people with different characteristics. Logistic regression analysis will be used to analyze the factors influencing chronic diseases among older people. A *p*-value of <0.05 was considered statistically significant.

## 3 Results

### 3.1 Basic information

For older people in HC, the mean age was (69.4 ± 7.4) years. There was a higher proportion of males (57.33%) than that of females (42.67%). The presence of marital history (married/divorced/widowed) was the highest percentage (98.20%). In addition, there are varying degrees of functional impairment among the older people. Specifically, 59.13, 28.53, and 6.17% of the older people had abnormal vision, sleep disorders, and swallowing disorders, respectively. See Table 1.

**Table 1:** The prevalence of chronic diseases among older people in HC with different characteristics.

Characteristics	Number of survey respondents (proportion, %)	Number of cases of chronic diseases (proportion, %)	χ^2^	*p*-value
Gender			1.145	0.285
Male	166 (42.67)	117 (70.48)		
Female	223 (57.33)	168 (75.34)		
Age (years)			1.131	0.288
60–79	344 (88.43)	255 (74.13)		
≥80	45 (11.57)	30 (66.67)		
Marital status			0.946	0.390
Never married	7 (1.80)	4 (57.14)		
Married/divorced/widowed	382 (98.20)	281 (73.56)		
Medical insurance			3.421	0.092
Without	12 (3.08)	6 (50.00)		
With	377 (96.92)	279 (74.01)		
Nationality			1.850	0.174
Han nationality	198 (50.90)	151 (76.26)		
Minority nationality	191 (49.10)	134 (70.20)		
Career			0.242	0.623
Farmer	277 (71.21)	201 (72.56)		
Non-farmer	112 (28.79)	84 (75.00)		
Education			2.146	0.543
Primary school and below	250 (64.27)	179 (71.60)		
Junior high school	79 (20.31)	62 (78.48)		
High school	36 (9.25)	25 (69.44)		
College degree or above	24 (6.17)	19 (79.17)		
Currently living alone			1.275	0.259
No	352 (90.49)	255 (72.44)		
Yes	37 (9.51)	30 (81.08)		
Time of day to be alone (hour, h)			9.222	0.022
<1	174 (44.73)	119 (68.39)		
1–2	45 (11.57)	29 (64.44)		
2–8	163 (41.90)	132 (80.98)		
>8	7 (1.80)	5 (71.43)		
BMI (kg/m^2^)			3.658	0.160
Low weight < 18.5	29 (7.46)	17 (58.62)		
Normal weight 18.5 ~ 23.9	180 (46.27)	132 (73.33)		
Overweight or obese >24.0	180 (46.27)	136 (75.56)		
Hearing			0.578	0.447
Normal	348 (89.46)	257 (73.85)		
Abnormal	41 (10.54)	28 (68.29)		
Vision			1.637	0.244
Normal	159 (40.87)	111 (69.81)		
Abnormal	230 (59.13)	174 (75.65)		
Balance ability			1.103	0.568
Normal	386 (99.23)	282 (73.06)		
Impaired	3 (0.77)	3 (100.00)		
Constipation			2.222	0.172
No	322 (82.78)	231 (71.74)		
Yes	67 (17.22)	54 (80.60)		
Sleep disorders			12.037	0.001
No	278 (71.47)	190 (68.35)		
Yes	111 (28.53)	95 (85.59)		
Smoked			0.034	0.904
No	259 (66.58)	189 (72.97)		
Yes	130 (33.42)	96 (73.85)		
Drank			0.521	0.542
No	262 (67.35)	189 (72.14)		
Yes	127 (32.65)	96 (75.59)		
Swallowing disorders			2.646	0.151
No	365 (93.83)	264 (72.33)		
Yes	24 (6.17)	21 (87.50)		
Primary mode of locomotion			2.224	0.349
Walking, no assistive device	383 (98.46)	279 (72.85)		
Walking, using an assistive device	6 (1.54)	6 (100.00)		
Self-rated health status			32.039	0.000
Very good	105 (26.99)	58 (55.24)		
Good	157 (40.36)	115 (73.25)		
Fair	123 (31.62)	108 (87.80)		
Poor	5 (1.29)	4 (80.00)		

BMI, Body Mass Index.

For older people in LTCFs, the mean age was (79.1 ± 9.1) years. Its gender proportion was similar to that of HC. The majority of them are farmers (67.33%). The presence of marital history was the highest percentage (78.71%). Moreover, 67.82% of the older people had medical insurance. In addition, 35.64%, 55.45%, 47.52%, 37.62%, and 26.24% of older people had abnormal hearing, abnormal vision, impaired balance, sleep disorders, and swallowing disorders, respectively. See Table 2.

**Table 2:** The prevalence of chronic diseases among older people in LTCFs with different characteristics.

Characteristics	Number of survey respondents (proportion, %)	Number of cases of chronic diseases (proportion, %)	χ^2^	*P*-value
Gender			0.908	0.398
Male	115 (56.93)	86 (74.78)		
Female	87 (43.07)	70 (80.46)		
Age (years)			2.634	0.105
60–79	93 (46.04)	67 (72.04)		
≥80	109 (53.96)	89 (81.65)		
Marital status			6.474	0.014
Never married	43 (21.29)	27 (62.79)		
Married/divorced/widowed	159 (78.71)	129 (81.13)		
Medical insurance			0.623	0.474
Without	65 (32.18)	48 (73.85)		
With	137 (67.82)	108 (78.83)		
Nationality			2.921	0.087
Han nationality	97 (48.02)	80 (82.47)		
Minority nationality	105 (52.00)	76 (72.38)		
Career			0.497	0.481
Farmer	136 (67.33)	107 (78.68)		
Non-farmer	66 (32.67)	49 (74.24)		
Education			1.51	0.681
Primary school and below	139 (68.81)	110 (79.14)		
Junior high school	41 (20.30)	29 (70.73)		
High school	17 (8.42)	13 (76.47)		
College degree or above	5 (2.48)	4 (80.00)		
Living alone before admission			0.391	0.615
No	96 (47.52)	76 (79.17)		
Yes	106 (52.48)	80 (75.47)		
BMI (kg/m^2^)			6.711	0.036
Low weight < 18.5	25 (12.38)	20 (80.00)		
Normal weight 18.5 ~ 23.9	108 (53.47)	76 (70.37)		
Overweight or obese >24.0	69 (34.16)	60 (86.96)		
Hearing			2.601	0.117
Normal	130 (64.36)	105 (80.77)		
Abnormal	72 (35.64)	51 (70.83)		
Vision			0.029	0.865
Normal	90 (44.55)	69 (76.67)		
Abnormal	112 (55.45)	87 (77.68)		
Balance ability			1.933	0.182
Normal	106 (52.48)	86 (81.13)		
Impaired	96 (47.52)	70 (72.92)		
Constipation			0	1
No	158 (78.22)	122 (77.22)		
Yes	44 (21.78)	34 (77.27)		
Sleep disorders			12.742	0.001
No	126 (62.38)	87 (69.05)		
Yes	76 (37.62)	69 (90.79)		
Smoked			1.143	0.285
No	127 (62.87)	95 (74.80)		
Yes	75 (37.13)	61 (81.33)		
Drank			0.67	0.413
No	144 (71.29)	109 (75.69)		
Yes	58 (28.71)	47 (81.03)		
Swallowing disorders			0.126	0.849
No	149 (73.76)	116 (77.85)		
Yes	53 (26.24)	40 (75.47)		
Primary mode of locomotion			10.459	0.012
Walking, no assistive device	108 (53.47)	76 (70.37)		
Walking, using an assistive device	60 (29.70)	50 (83.33)		
Wheelchair	25 (12.38)	24(96.00)		
Bedbound	9 (4.46)	6 (66.67)		
Self-rated health status			104.858	0.001
Very good	37 (13.51)	5 (13.51)		
Good	76 (93.42)	71 (93.42)		
Fair	78 (89.74)	70 (89.74)		
Poor	11 (90.91)	10 (90.91)		

BMI, Body Mass Index.

### 3.2 Current status of chronic diseases

The prevalence of chronic diseases among older people in HC was 73.26% (95% CI, 68.85–77.68), and 41.39% of older people suffered from two or more chronic diseases. The top five chronic diseases were hypertension (26.36%), bone and joint disease (23.36%), gastrointestinal and gallbladder disease (11.78%), heart disease (11.21%), and diabetes (8.97%). See Table 3.

**Table 3:** The prevalence of chronic diseases among older people in HC and LTCFs.

HC		LTCFs	
Chronic diseases	Prevalence (%)	Chronic diseases	Prevalence (%)
Hypertension	26.36	Hypertension	33.11
Bone and joint disease	23.36	Bone and joint disease	13.25
Gastrointestinal gallbladder disease	11.78	Cerebrovascular disease	12.91
Heart disease	11.21	Diabetes	11.26
Diabetes	8.97	Heart disease	10.26
Chronic respiratory diseases	6.36	Gastrointestinal gallbladder disease	6.62
Cerebrovascular disease	4.86	Cataract	4.30
Hyperlipidemia	2.80	Chronic respiratory diseases	2.98
Cataract	2.06	Benign prostatic hyperplasia	1.66
Gout	1.12	Parkinson’s disease	1.66
Cancer	0.37	Hyperlipidemia	0.66
Anemia of chronic disease	0.37	Myasthenia gravis	0.33
Uremia	0.37	Anemia of chronic disease	0.33
		Leukemia	0.33
		Uremia	0.33

The prevalence of chronic diseases among older people in LTCFs was 77.23% (95% CI, 77.23–83.06), and 43.56% of older people suffered from two or more chronic diseases. The top five chronic diseases were hypertension (33.11%), bone and joint disease (13.25%), cerebrovascular disease (12.91%), diabetes (11.26%), and heart disease (10.26%). See Table 3.

### 3.3 One-way analysis of variance

The results of the one-way analysis of variance showed that the time of day to be alone, sleep disorders, and self-rated health status of the older people in HC with chronic diseases were statistically significant (*p* < 0.05). See Table 1.

For the older people in LTCFs with chronic diseases, it showed that the marital status, BMI, sleep disorders, primary mode of locomotion, and self-rated health status of the older people in LTCFs with chronic diseases were statistically significant (*p* < 0.05). See Table 2.

### 3.4 Chronic disease influencing factor model

Chronic disease influencing factor models are developed based on the above investigation and logistic regression analysis. For the older people in HC with chronic diseases, it shows that long time spent alone, having sleep disorders, and self-rated health status significantly increased the prevalence of chronic diseases (*p* < 0.05). As older people spend more time alone throughout the day, their risk of chronic diseases increases more significantly. The prevalence of chronic diseases in older people who spend 2–8 h and > 8 h alone are 1.968 and 1.155 times than that of those who spend <1 h alone. The prevalence of chronic diseases in older people with sleep disorders is 2.750 times than that of those without sleep disorders. Besides, the prevalence of older people who have good, fair, and poor self-rated health status are 2.219, 6.251, and 3.241 times than that of those who have very good self-rated health status. See Table 4.

**Table 4:** Logistic regression analysis of chronic diseases in older people in HC.

Characteristics	Eigenvalue	Reference group	β	SE	Wald	*P*-value	OR	95% CI
Time of day to be alone (hour, h)	1–2	<1	−0.177	0.352	0.254	0.614	0.838	0.421 ~ 1.668
	2–8		0.677	0.258	6.901	0.009	1.968	1.188 ~ 3.261
	>8		0.145	0.852	0.029	0.865	1.155	0.217 ~ 6.142
Sleep disorders	Yes	No	1.012	0.299	11.414	0.001	2.750	1.529 ~ 4.945
Self-rated health status	Good	Very good	0.797	0.267	8.943	0.003	2.219	1.316 ~ 3.741
	Fair		1.833	0.345	28.179	0	6.251	3.177 ~ 12.299
	Poor		1.176	1.135	1.073	0.3	3.241	0.350 ~ 29.988

For the older people in LTCFs with chronic diseases, it shows that having marital status, non-healthy BMI, having sleep disorder, walking with the use of assistive devices, and self-rated health status significantly increased their prevalence (*p* < 0.05). The prevalence of chronic diseases of older people who are married/divorced/widowed is 2.548 times than that of those who have never been married. Furthermore, the risk of chronic diseases in older people also increases as BMI decreases, reaching a level of 0.600 times. The prevalence of chronic diseases in older people with sleep disorders is 4.419 times than that of those without sleep disorders. Among the primary modes of locomotion, the prevalence of chronic diseases of older people who walk with the use of assistive devices and wheelchairs are 2.105 and 10.105 times than that of those who walk without assistive devices. Besides, the prevalence of older people who have good, fair, and poor self-rated health status are 90.880, 56.000, and 64.000 times than that of those who have very good self-rated health status. See Table 5.

**Table 5:** Logistic regression analysis of chronic diseases in older people in LTCFs.

Characteristic	Eigenvalue	Reference group	β	SE	Wald	*P*-value	OR	95% CI
Marital status	Married/divorced/widowed	Never married	0.935	0.375	6.222	0.013	2.548	1.222 ~ 5.314
BMI (kg/m^2^)	<18.5	>24.0	−0.511	0.615	0.691	0.406	0.600	0.180 ~ 2.001
	18.5–23.9		−1.032	0.415	6.187	0.013	0.356	0.158 ~ 0.803
Sleep disorders	Yes	No	1.486	0.441	11.352	0.001	4.419	1.862 ~ 10.488
Primary mode of locomotion	Walking, using an assistive device	Walking, no assistive device	0.744	0.405	3.371	0.066	2.105	0.951 ~ 4.661
	Wheelchair		2.313	1.042	4.926	0.026	10.105	1.311 ~ 77.916
	Bedbound		−0.172	0.738	0.054	0.816	0.842	0.198 ~ 3.576
Self-rated health status	Good	Very good	4.510	0.667	45.664	0.000	90.880	24.572 ~ 336.127
	Fair		4.025	0.609	43.730	0.000	56.000	16.984 ~ 184.641
	Poor		4.159	1.154	12.993	0.000	64.000	6.669 ~ 614.179

BMI, Body Mass Index.

## 4 Discussion

In this study, the prevalence of chronic diseases among older people in HC and LTCFs was higher than 70.00%. This is significantly higher than that of developed countries, such as the United States (60.00%) (26), Germany (62.10%) (27), and Australia (66.30%) (28), and lower than that of in six low- and middle-income countries (80.59%) (29). These results demonstrate that the prevalence of chronic diseases is higher in low-income and middle-income countries than in developed countries (30). This is the result of complex social, economic, and behavioral factors at different development levels in various countries (31). In these two different aged care models, hypertension, bone and joint disease, heart disease, and diabetes were the most prevalent chronic diseases among older people in HC and LTCFs. Bone and joint diseases are caused by the mountainous areas where part of the respondents reside (32).

The prevention and treatment of chronic diseases in older people are more important to pay attention to risk factors because of the shortage of medical and care resources in China. In this study, one-way analysis of variance shows that the time of day to be alone, sleep disorders, and self-rated health status were the main risk influences on the prevalence of chronic diseases among older people in HC vs. marital status, BMI, sleep disorders, primary mode of locomotion, and self-rated health status for older people in LTCFs. People who are married/divorced/widowed are more likely to suffer from chronic diseases than those who have never been married, which is due to the heavy family and social responsibilities of older people with a marriage history (33–35). In addition, Qian (36) found that older people who sleep long at night and do not sleep during the day are more likely to suffer from diabetes. In this study, the association between sleep disorders and chronic disease is further confirmed, with sleep disorders significantly increasing the prevalence of chronic disease in older people. Besides, high prevalence of chronic diseases in older people who walk with the use of assistive devices and spend extensive periods alone. This is attributed to the decline of physiological functions as a result of aging in older people, causing them to exercise less and increasing their risk of developing chronic diseases (37). Besides, their long time alone and limited family support cause them more risk of chronic illnesses, both physically and mentally (38, 39).

The above studies prove that HC and LTCFs have different risk factors for chronic diseases in older people. Thus, older people under different aged care models need to propose corresponding healthcare strategies based on their characteristics. Further summary found that older people in HC spend a lot of time alone and then suffer from loneliness, which ultimately causes psychological disorders. Thus, psychological adaptation interventions are needed for older people in HC, such as psychotherapy, psychological counseling, psychological crisis intervention, and so on. Besides, older people in LTCFs lack social support from their families (divorced/widowed) and have activity disorders (walking with the use of assistive devices). Thus, social adaptation interventions are needed for older people in LTCFs, such as health education, changing poor lifestyles, providing social support, and so on. The above healthcare strategies are obtained through the chronic disease influencing factor model. This care strategy not only improves the quality of life of older people in two different aged care models but also optimizes the distribution of care resources between the two different aged care models.

This study used a cross-sectional study, which is one of the best ways to measure the prevalence of health outcomes and can study the associations between multiple exposures and outcomes (40). Nevertheless, it has some limitations. The cross-sectional study design limited the ability to infer causal relationships from the findings. Furthermore, cohort studies are required to determine causal relationships. Second, as the data are self-reported, they may influence participants’ responses and lead them to overestimate their abilities. In future studies, more objective indicators should be added.

## 5 Conclusion

There are differences in the prevalence and distribution of chronic diseases among older people in two different aged care models in China. Moreover, there are various risk factors for chronic diseases. Therefore, chronic disease healthcare strategies should be tailored to two different aged care models for older people. Further summary found that older people in HC spend a lot of time alone and suffer from loneliness, which ultimately causes psychological disorders. Thus, psychological adaptation interventions are needed for older people in HC. Besides, older people in LTCFs lack social support from their families (divorced/widowed) and have activity disorders (walking with the use of assistive devices). Thus, social adaptation interventions are needed for older people in LTCFs. This study provides a theoretical basis for the distribution of healthcare and the prevention and treatment of chronic diseases in Chinese older people. It also can be considered as a starting point for further care needs and intervention research studies in chronic diseases among older people.

## Data availability statement

The original contributions presented in the study are included in the article/supplementary material, further inquiries can be directed to the corresponding author/s.

## Ethics statement

The studies involving humans were approved by Biomedical Ethics Committee of Jishou University (JSDX-2021-0027). The studies were conducted in accordance with the local legislation and institutional requirements. Written informed consent for participation in this study was provided by the participants’ legal guardians/next of kin.

## Author contributions

FX: Funding acquisition, Writing – original draft, Writing – review & editing. QS: Formal analysis, Investigation, Writing – review & editing. JL: Funding acquisition, Investigation, Writing – review & editing. Z-yC: Formal analysis, Writing – review & editing.
